# Analysis of Published Criteria for Clinically Inactive Disease in a Large Juvenile Dermatomyositis Cohort Shows That Skin Disease Is Underestimated

**DOI:** 10.1002/art.39200

**Published:** 2015-08-26

**Authors:** Beverley Almeida, Raquel Campanilho‐Marques, Katie Arnold, Clarissa A. Pilkington, Lucy R. Wedderburn, Kiran Nistala, on behalf of the Juvenile Dermatomyositis Research Group, Kate Armon, Vanja Briggs, Joe Ellis‐Gage, Holly Roper, Joanna Watts, Eileen Baildam, Louise Hanna, Olivia Lloyd, Liza McCann, Ian Roberts, Ann McGovern, Phil Riley, Eslam Al‐Abadi, Clive Ryder, Janis Scott, Taunton Southwood, Beverley Thomas, Tania Amin, Deborah Burton, Gillian Jackson, Vanessa Van Rooyen, Mark Wood, Sue Wyatt, Michael Browne, Joyce Davidson, Sue Ferguson, Janet Gardner‐Medwin, Neil Martin, Liz Waxman, Helen Foster, Mark Friswell, Sharmila Jandial, Lisa Qiao, Ethan Sen, Eve Smith, Vicky Stevenson, Alison Swift, Debbie Wade, Stuart Watson, Lindsay Crate, Anna Frost, Mary Jordan, Ellen Mosley, Rangaraj Satyapal, Elizabeth Stretton, Helen Venning, Kishore Warrier, Beverley Almeida, Katie Arnold, Laura Beard, Virginia Brown, Raquel Campanilho‐Marques, Elli Enayat, Yvonne Glackin, Elizabeth Halkon, Nathan Hasson, Audrey Juggins, Laura Kassoumeri, Sian Lunt, Sue Maillard, Kiran Nistala, Clarissa Pilkington, Stephanie Simou, Sally Smith, Hemlata Varsani, Lucy Wedderburn, Kevin Murray, John Ioannou, Linda Suffield, Muthana Al‐Obaidi, Sam Leach, Helen Lee, Helen Smith, Emma Inness, Eunice Kendall, David Mayers, Nick Wilkinson, Jacqui Clinch, Helen Pluess‐Hall

**Affiliations:** ^1^ Great Ormond Street Hospital for Children, NHS Trust and University College LondonLondonUK; ^2^ University College LondonLondonUK; ^3^ Great Ormond Street Hospital for Children, NHS TrustLondonUK; ^4^ University College London, University College London Hospital, and Great Ormond Street Hospital for Children, NHS TrustLondonUK

## Abstract

**Objective:**

The Pediatric Rheumatology International Trials Organisation (PRINTO) recently published criteria for classification of patients with juvenile dermatomyositis (DM) as having clinically inactive disease. The criteria require that at least 3 of 4 conditions be met, i.e., creatine kinase level ≤150 units/liter, Childhood Myositis Assessment Scale score ≥48, Manual Muscle Testing in 8 muscles score ≥78, and physician's global assessment of overall disease activity (PGA) ≤0.2. The present study was undertaken to test these criteria in a UK cohort of patients with juvenile DM.

**Methods:**

We assessed 1,114 patient visits for the 4 items in the PRINTO criteria for clinically inactive disease. Each visit was analyzed to determine whether skin disease was present. The Disease Activity Score (DAS) for juvenile DM was determined in 59 patients.

**Results:**

At 307 of the 1,114 visits, clinically inactive disease was achieved based on the 3 muscle criteria (but with a PGA of >0.2); rash was present at 65.8% of these visits and nailfold capillary abnormalities at 35.2%. When PGA ≤0.2 was one of the 3 criteria that were met, the frequency of skin signs was significantly lower (rash in 23.1% and nailfold capillary abnormalities in 8.7%). If PGA was considered an essential criterion for clinically inactive disease (P‐CID), patients with active skin disease were less likely to be categorized as having clinically inactive disease (a median DAS skin score of 0 [of a possible maximum of 9] in visits where the PGA was ≤0.2, versus a median DAS skin score of 4 in patients meeting the 3 muscle criteria [with a PGA of >0.2]; *P* < 0.001). Use of the P‐CID led to improvements in the positive predictive value and the positive likelihood ratio (85.4% and 11.0, respectively, compared to 72.9% and 5.1 with the current criteria).

**Conclusion:**

There was a high frequency of skin disease among patients with juvenile DM who did not meet the PGA criterion for inactive disease but met the other 3 criteria. Incorporating PGA as an essential criterion for clinically inactive disease helps prevent the misclassification of patients with active skin disease.

Juvenile dermatomyositis (DM) affects approximately 2–3 children/million/year, and although rare, is the most common childhood form of idiopathic inflammatory myopathy 1, 2. Scoring tools have been developed to assess disease activity and damage in juvenile DM in a standardized manner 3, to assist in the conduct of clinical trials and allow comparisons between different cohorts. The Pediatric Rheumatology International Trials Organisation (PRINTO) recently analyzed these activity measures and defined thresholds for classification of clinically inactive disease 4. Disease was considered clinically inactive if the patient met at least 3 of 4 criteria, i.e., creatine kinase (CK) level ≤150 units/liter, Childhood Myositis Assessment Scale (CMAS) score 5 ≥48, Manual Muscle Testing in 8 muscles (MMT‐8) score 6 ≥78, and physician's global assessment of overall disease activity (PGA) ≤0.2.

These criteria are currently weighted toward muscle disease. Although muscle symptoms are the main focus of monitoring and treatment in juvenile DM, it is important that skin inflammation is not neglected. Skin disease is often resistant to treatment and may be associated with poor long‐term outcomes such as calcinosis 7, 8, poor quality of life, and reduced physical function 9, 10. Therefore, we propose that skin disease should be represented in any definition of clinically inactive disease. At present, a patient with juvenile DM must meet only 3 of 4 criteria to be classified as having clinically inactive disease. Therefore, if all 3 muscle criteria are met, the PGA may be disregarded. This poses the potential risk that disease activity in the skin or other organs will be ignored. The purpose of this study was to apply the PRINTO criteria for clinically inactive disease to a UK cohort of patients with juvenile DM and test the hypothesis that in clinical practice, there may be alternative definitions that would improve the performance of the criteria.

## PATIENTS AND METHODS

### Patients

The study population consisted of patients from the UK Juvenile Dermatomyositis Cohort and Biomarker Study 11; 1,114 discrete visits involving 258 patients were analyzed. Written informed consent was obtained from the legal guardians of all patients. All patients met the Bohan and Peter criteria for diagnosis of juvenile DM 12, 13; 74.6% were female and 80.9% were white. The mean ± SD age at the time of the visit assessed for the present study was 11.9 ± 3.6 years. The mean age at diagnosis was 6.7 ± 3.4 years, and the mean disease duration was 4.4 ± 3.1 years.

### Data collection

Patient clinical data were collected at the time of recruitment and then prospectively every 3–4 months for the first 2 years and subsequently, at least once a year. Data collected included signs and symptoms and disease activity measures: CMAS, MMT‐8, PGA, and laboratory tests. Data related to skin disease (rash, Gottron's papules, ulceration, nailfold changes, calcinosis) were retrieved for all patient visits. All data for the UK Juvenile Dermatomyositis Cohort and Biomarker Study are stored in a Structured Query Language platform database with Access front‐end data retrieval. Research coordinators and principal investigators at the local study centers are listed in Appendix A.

Fifty‐nine of the patients were clinically assessed using the Disease Activity Score (DAS) for juvenile DM instrument 14. The DAS was determined by 1 of 2 physicians (BA and RC‐M) at the time of clinical assessment of the patient. The DAS instrument consists of 6 components, resulting in a 20‐point scale, with higher scores indicating greater disease activity. It has 2 subsections, the DAS muscle score (scored 0–11) addressing functional status and the presence or absence of weakness, and the DAS skin score (scored 0–9) related to skin disease including skin involvement type, distribution, vasculitis, and Gottron's papules. For the purposes of the present study, the DAS instrument was applied in its entirety (score 0–20), and separated into its muscle and skin subsections.

### Data analysis

Patient visits were included in the study if data on all 4 of the PRINTO criteria for clinically inactive disease 4 were available. Clinically inactive disease could be designated if all 4 criteria were met or if only 3 of the 4 were met. On this basis, patient visits were divided into groups (Table 1).

**Table 1:** Juvenile DM groups according to disease activity and PRINTO criteria met^a^

Group	Disease status	No. of PRINTO criteria met	PGA	Other criteria
I	Clinically inactive	4	Met (score ≤0.2)	All met
II	Clinically inactive	3	Met (score ≤0.2)	Either CK or CMAS or MMT‐8 not met
III	Clinically inactive	3	Not met (score >0.2)	CK, CMAS, and MMT‐8 all met
IV	Active	0 or 1	NA	NA

a PRINTO criteria = Pediatric Rheumatology International Trials Organisation criteria for clinically inactive juvenile dermatomyositis (DM); PGA = physician's global assessment of overall disease activity; CK = creatine kinase; CMAS = Childhood Myositis Assessment Scale; MMT‐8 = Manual Muscle Testing in 8 muscles; NA = not applicable.

### Statistical analysis

Normally distributed continuous variables were reported as the mean ± SD, and non‐normally distributed continuous variables as the median and range. Categorical data were analyzed by chi‐square test. One‐way analysis of variance (ANOVA) with Tukey's correction for multiple testing was used to test the significance of differences in DAS scores between groups. *P* values less than 0.05 were considered significant.

Using diagnostic statistics, we compared the performance of the original criteria against “P‐CID,” an alternative definition of clinically inactive disease in which PGA is regarded as an essential criterion together with either 2 of the 3 muscle criteria. From the original cohort of 1,114 patient visits, both definitions of clinically inactive disease were tested using a reference group of patient visits: those occurring within 4 months of diagnosis and with the patient receiving medication (active), and those occurring when the patient had not received any medication for ≥6 months (inactive, or clinical remission as defined by the International Myositis Assessment and Clinical Studies Group consensus guidelines [15]). The time frame of 4 months from diagnosis was used based on our assumption that these patients would have active disease. In order to demonstrate whether P‐CID would improve the performance of the criteria for clinically inactive disease, separate analyses including sensitivity, specificity, positive and negative predictive values, and positive and negative likelihood ratios (with corresponding 95% confidence intervals) were performed.

Data were stored in a central Access database and analyzed with Excel. GraphPad Prism version 5.00 for Windows was used for statistical analyses.

## RESULTS

**Figure 1:**
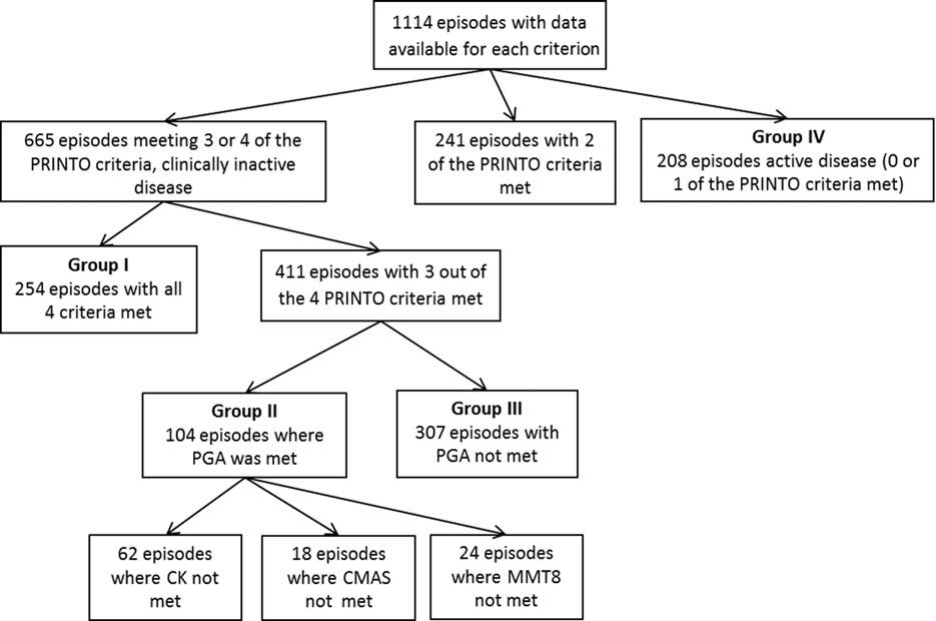
Flow chart of 1,114 visits among 258 patients from the UK Juvenile Dermatomyositis Cohort and Biomarker Study, according to the Pediatric Rheumatology International Trials Organisation (PRINTO) criteria for clinically inactive disease. PGA = physician's global assessment of overall disease activity; CK = creatine kinase; CMAS = Childhood Myositis Assessment Scale; MMT‐8 = Manual Muscle Testing in 8 muscles.

Among our cohort of 1,114 visits in 258 patients with juvenile DM, the criteria for clinically inactive disease were met at 665 visits (59.7%) (Figure 1). All 4 of the criteria were met at 254 (38.2%) of these 665 visits (in 119 patients) (group I), while 3 of the 4 criteria were met at the remaining 411 visits (61.8%) (in 165 patients). Of the visits at which only 3 of the criteria were met, the PGA was ≤0.2 in 104 (group II), while at the remaining 307, disease was clinically inactive based on the 3 muscle criteria, but the PGA was >0.2 (group III).

**Figure 2:**
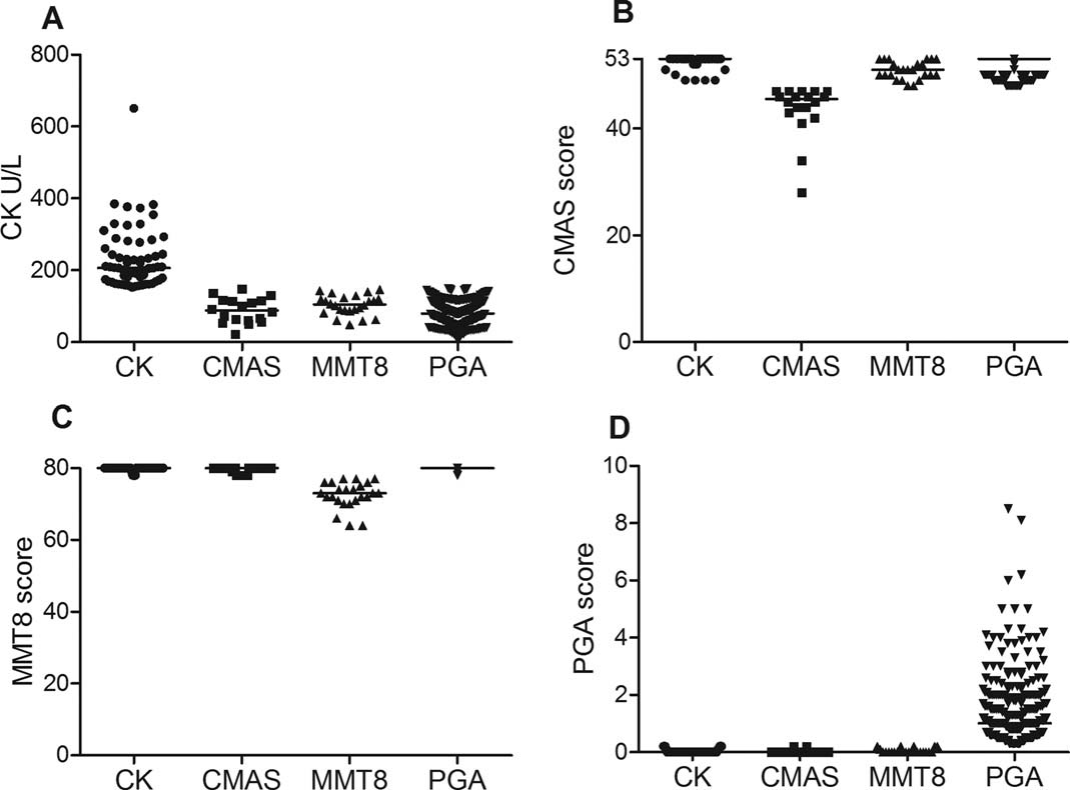
Analysis of disease activity and laboratory results in the 411 patient visits at which 3 of the 4 criteria for clinically inactive disease were met. Each panel shows data analyzed when 1 of the 4 criteria was not met (i.e., CK in **A,** CMAS in **B,** MMT‐8 in **C,** and PGA in **D**). Each symbol represents an individual visit; horizontal lines show the median. See Figure 1 for definitions.

To test if each of the criteria were equally redundant, the 411 visits were divided based on which of the criteria for clinically inactive disease was not met. The median CK value in the group with CK >150 units/liter was 206 units/liter (Figure 2A), and values were <400 units/liter in the majority of outliers (range 152–650). CMAS scores in the group not meeting the CMAS threshold were clustered about a median value of 45, although values of 28 and 34 were noted in the case of 2 outliers (Figure 2B). For patient visits not meeting the MMT‐8 threshold, scores were clustered close to 78 (median 73 [range 71–77]) (Figure 2C). However, for PGA, unlike the findings for CK, CMAS, and MMT‐8, it was striking that the distribution of scores when the PGA was >0.2 spanned the entire spectrum, from 0.3 to 8.5 (median 1.0) (Figure 2D).

### Frequency of skin disease

In clinical practice, the most common reason for ongoing juvenile DM disease activity in the setting of normal muscle results is skin inflammation. To address this, the frequency of skin signs was analyzed according to which of the specific criteria for clinically inactive disease were met (Table 2). It is generally thought by clinicians that a PGA score of >1.0 represents active disease. Of the visits in group III, the PGA was >1.0 in 44% despite all of the muscle‐related criteria being met, suggesting the presence of ongoing disease activity that did not involve muscles.

**Table 2:** Frequency of skin signs in patients with juvenile DM meeting the PRINTO criteria for clinically inactive disease^a^

	Rash	Gottron's papules	Ulceration	Nailfold changes	Calcinosis
Group I (254 visits, 118 patients)	76 (29.9)	32 (12.6)	1 (0.4)	28 (11.0)	19 (7.5)
Group II (104 visits, 70 patients)	24 (23.1)	11 (10.6)	1 (1.0)	9 (8.7)	6 (5.8)
Group III (307 visits, 131 patients)	202 (65.8)	123 (40.1)	9 (2.9)	108 (35.2)	60 (19.5)
Group IV (208 visits, 127 patients)	159 (76.4)	117 (56.3)	26 (12.5)	100 (48.1)	36 (17.3)
χ^2^ (*P*)					
Group I vs. group II	1.71 (NS)	0.29 (NS)	0.43 (NS)	0.45 (NS)	0.33 (NS)
Group I vs. group III	71.57 (<0.0001)	52.44 (<0.0001)	5.11 (0.024)	44.16 (<0.0001)	16.72 (0.0009)
Group II vs. group III	57.28 (<0.0001)	30.74 (<0.0001)	1.27 (NS)	26.84 (<0.0001)	10.93 (0.0009)

a See Table 1 for description of groups I, II, III, and IV. Values are the number (%) of visits. NS = not significant (see Table 1 for other definitions).

In group I (meeting all 4 of the criteria), skin signs were still present, with rash observed in almost 30% and nailfold abnormalities in 11% of the visits. Group II had no significant differences in skin abnormalities when compared to group I. In group III, the frequencies of skin signs were much higher and the data closely paralleled results in group IV. In group III (the PGA criterion not met), the frequency of skin signs (except for ulceration) was significantly increased compared to group II (for rash, χ^2^ = 57.28, *P* < 0.0001; for Gottron's papules, χ^2^ = 30.74, *P* < 0.0001; for nailfold changes, χ^2^ = 26.84, *P* < 0.0001; for calcinosis, χ^2^ = 10.93, *P* = 0.0009). There were no significant differences in the frequency of ulceration.

### Scores on the DAS for juvenile DM

**Figure 3:**
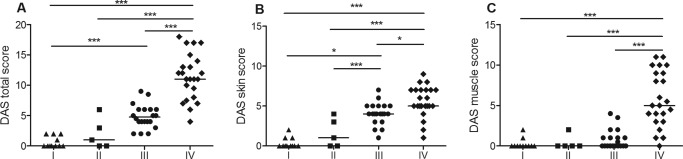
Scores according to the Disease Activity Score (DAS) for juvenile dermatomyositis in 59 patients meeting criteria for clinically inactive disease. DAS total score (**A**), DAS skin score (**B**), and DAS muscle score (**C**) in patients who met all 4 criteria for clinically inactive disease (group I), patients who met 3 of the 4 criteria, including the physician's global assessment (PGA) ≤0.2 criterion (group II), patients who met 3 of the 4 criteria but not including the PGA ≤0.2 criterion (group III), and patients who met 0 or 1 of the criteria (active disease [group IV]) are shown. Each symbol represents an individual patient; horizontal lines show the median. ∗ = *P* < 0.05; ∗∗∗ = *P* < 0.001.

Our results suggested that PGA was an important criterion since it identified ongoing skin disease. To confirm this hypothesis, we used the DAS 14 to perform a detailed analysis of muscle and skin disease in 59 of the patients with juvenile DM. As expected, DAS total scores were low in group I (median 0 [range 0–2]) (Figure 3A), suggesting that the combined criteria successfully excluded patients with active disease. There was no significant increase in DAS total scores when group II (median 1 [range 0–6]) was compared to group I (the reference group). However, the DAS total score in group III was significantly increased (median 5 [range 2–9]; *P* < 0.01 by ANOVA).

To further explore the role of PGA within the set of criteria for clinically inactive disease, we reanalyzed the above data after separating the DAS total score into its skin and muscle subsections. The DAS skin score (Figure 3B) mirrored the DAS total score in that there were no significant differences between group I (median 0 [range 0–2]) and group II (median 0 [range 0–4]). DAS skin scores in group III (median 4 [range 1–7]) were significantly worse than those in groups I and II. For the DAS muscle score, in contrast, there were no significant differences in between groups I, II, and III (median 0 in all 3 groups) (Figure 3C), confirming that there was almost no active muscle disease.

### Diagnostic performance of revised criteria

The above results suggest that an increased PGA in the context of normal muscle findings identifies patients with juvenile DM who have ongoing skin inflammation. As the P‐CID appeared more stringent than the original PRINTO criteria (Table 2), we wondered if this would reduce its diagnostic utility. We thus compared the performance of the original criteria against the P‐CID (i.e., with PGA ≤0.2 as an essential criterion, together with either 2 or 3 of the muscle‐related criteria). Both definitions of clinically inactive disease were tested, using the originally identified 1,114 patient visits, to create 2 reference groups: 1) active disease (defined as patients who had been diagnosed ≤4 months previously and were taking medication) (total 111 visits), and 2) inactive disease (defined as patients who had not been taking medications for ≥6 months) (total 59 visits).

We applied the PRINTO criteria to these 2 groups and classified the patient visits according to the number of criteria met. Among the 111 visits at which the disease was classified as active based on a duration of ≤4 months since diagnosis, 20 met the PRINTO criteria for clinically inactive disease. In 13 of the 20 visits, the PGA was >0.2 (3 criteria met); at 2 visits the PGA was ≤0.2 (3 criteria met), and all 4 of the criteria were met in 5 visits. Among the 59 visits at which the disease was classified as inactive based on a period of ≥6 months since treatment, 54 met the PRINTO criteria for clinically inactive disease. At 13 of these 54 visits the PGA was >0.2 (3 criteria); at 11 visits the PGA was ≤0.2 (3 criteria), and all 4 of the criteria were met in 30 visits.

Consequently, we calculated sensitivity, specificity, positive and negative predictive values, and positive and negative likelihood ratios based on the current PRINTO criteria and the P‐CID (Table 3). Compared to the current criteria, the use of the P‐CID led to an improvement in the positive predictive value (85.4% versus 72.9%) and positive likelihood ratio (11.0 versus 5.1), without an appreciable deterioration in the negative predictive value or negative likelihood ratio. Specificity also increased, to 93.7% (compared to 81.9% with the current criteria); however, sensitivity of the P‐CID was lower than that of the current criteria (69.5% versus 91.5%).

**Table 3:** Performance characteristics of the current (PRINTO) and revised criteria for clinically inactive juvenile DM^a^

	Current criteria	95% CI	Alternative definition of clinically inactive disease (P‐CID)	95% CI
Sensitivity, %	91.5	80.5–96.8	69.5	56.0–80.5
Specificity, %	81.9	73.3–88.3	93.7	87.0–97.2
PPV, %	72.9	61.1–82.3	85.4	71.6–93.5
NPV, %	94.8	87.7–98.0	85.2	77.4–90.8
PLR	5.1	3.4–7.6	11.0	5.3–23.0
NLR	0.1	0.04–0.2	0.3	0.2–0.5

a 95% CI = 95% confidence interval; PPV = positive predictive value; NPV = negative predictive value; PLR = positive likelihood ratio; NLR = negative likelihood ratio; P‐CID = clinically inactive disease when the PGA criterion is met (see Table 1 for other definitions).

## DISCUSSION

Many patients with juvenile DM have prolonged disease courses and require long‐term treatment. It is therefore important to be able to accurately define clinically inactive disease in order to aid in assessment of their condition and guide treatment decisions. To this end, PRINTO has proposed a set of criteria for clinically inactive disease in juvenile DM, which are based on disease activity measures that are in routine clinical use. In the present study we formally tested these criteria in a large independent cohort of patients with juvenile DM and investigated whether they performed adequately in a real‐world clinical setting.

Based on the PRINTO criteria, the definition of clinically inactive disease was met at nearly 60% of our patient visits. As 3 of the 4 PRINTO measures are specific to muscle disease, we wondered if there was redundancy between these items. Indeed, we found that omission of 1 of the 3 muscle criteria had little impact on the disease activity scores in that domain. In contrast, during visits at which patients met the 3 muscle criteria but not the PGA criterion, PGA scores were increased, despite normal muscle findings. Our results identified a subset of patients, with high PGA scores but normal muscle findings, who exhibited a high frequency of skin abnormalities. We predicted that these patients had active skin disease, but as our longitudinal cohort study data could not easily distinguish between damage and active disease (e.g., atrophic Gottron's papules), we prospectively assessed 59 patients using the DAS for juvenile DM. In this carefully characterized patient cohort, it was clear that patients in group III had significantly higher DAS skin scores than patients in group I or II, confirming that use of muscle criteria alone failed to identify patients with active skin disease.

Although the majority of the DAS instrument specifically identifies clinical activity, a few of the skin items may detect damage (e.g., atrophic changes within the skin involvement section of the instrument and telangiectasia in the vasculitis section). However, when these items were removed from the analysis, the results were unchanged (data not shown).

Recently, investigators from Norway retrospectively analyzed an inception cohort of 59 patients with juvenile DM who had been followed up for a long period of time 16. They found a rate of clinically inactive disease of 49%, similar to our results. Only 48% of the Norwegian patients meeting criteria for clinically inactive disease had a normal score on the Myositis Intention‐to‐Treat Activity Index (MITAX) (17), an instrument measuring disease activity in 7 distinct organ domains. However, if the skin domain was excluded from the MITAX, 87% of patients meeting criteria for clinically inactive disease had a normal score. These results are consistent with our finding that skin disease is underestimated with the use of the PRINTO criteria for clinically inactive disease.

One limitation of our study is that we did not have accurate data on disease activity in other organs. Although we show that many patients with a high PGA score in the setting of normal muscle results have active skin disease, it is possible that the elevated PGA score also relates to disease in other organs.

When compared to the existing PRINTO criteria, the use of the P‐CID improved the specificity and the positive predictive value for clinically inactive disease but reduced sensitivity, suggesting that our modification increased the stringency of the tool. In this analysis we considered visits occurring within 4 months of diagnosis to be representative of patients with clinically active disease. However, this may underestimate the specificity of tools for defining clinically inactive disease, as a small number of patients within this group had no evidence of active disease and may reflect a subset with a milder disease course or those who are early responders to treatment.

We also considered the use of the PGA and MMT‐8 alone to define clinically inactive disease, but this did not yield better results than our proposed P‐CID. However, such a 2‐item instrument warrants consideration in a further validation study examining criteria for clinically inactive disease.

We therefore propose that the existing PRINTO criteria for defining clinically inactive disease in patients with juvenile DM require modification, either with the use of the PGA as an essential criterion or by adding items that specifically measure skin disease activity. These modifications need to be tested in future studies, and should ensure that active skin disease related to juvenile DM is not overlooked in the definition of clinically inactive disease.

## AUTHOR CONTRIBUTIONS

All authors were involved in drafting the article or revising it critically for important intellectual content, and all authors approved the final version to be published. Dr. Nistala had full access to all of the data in the study and takes responsibility for the integrity of the data and the accuracy of the data analysis.

### Study conception and design

Almeida, Campanilho‐Marques, Pilkington, Wedderburn, Nistala.

### Acquisition of data

Almeida, Campanilho‐Marques, Pilkington, Wedderburn, Nistala.

### Analysis and interpretation of data

Almeida, Campanilho‐Marques, Arnold, Nistala.

## LOCAL PRINCIPAL INVESTIGATORS, RESEARCH COORDINATORS, AND MEMBERS OF THE JUVENILE DERMATOMYOSITIS RESEARCH GROUP

Contributing local research coordinators, principal investigators, and Juvenile Dermatomyositis Research Group members (all in the UK except as indicated otherwise) were as follows: Dr. Kate Armon, Ms Vanja Briggs, Mr. Joe Ellis‐Gage, Ms Holly Roper, Ms Joanna Watts (Norfolk and Norwich University Hospitals, Norwich); Dr. Eileen Baildam, Ms Louise Hanna, Ms Olivia Lloyd, Dr. Liza McCann, Mr. Ian Roberts (Royal Liverpool Children's Hospital, Liverpool); Ms Ann McGovern, Dr. Phil Riley (Royal Manchester Children's Hospital, Manchester); Dr. Eslam Al‐Abadi, Dr. Clive Ryder, Ms Janis Scott, Dr. Taunton Southwood, Ms Beverley Thomas (Birmingham Children's Hospital, Birmingham); Dr. Tania Amin, Ms Deborah Burton, Ms Gillian Jackson, Dr. Vanessa Van Rooyen, Dr. Mark Wood, Dr. Sue Wyatt (Leeds General Infirmary, Leeds); Mr. Michael Browne, Dr. Joyce Davidson, Ms Sue Ferguson, Dr. Janet Gardner‐Medwin, Dr. Neil Martin, Ms Liz Waxman (Royal Hospital for Sick Children, Glasgow); Dr. Helen Foster, Dr. Mark Friswell, Dr. Sharmila Jandial, Ms Lisa Qiao, Dr. Ethan Sen, Dr. Eve Smith, Ms Vicky Stevenson, Ms Alison Swift, Ms Debbie Wade, Mr. Stuart Watson (Great North Children's Hospital, Newcastle); Ms Lindsay Crate, Ms Anna Frost, Ms Mary Jordan, Dr. Ellen Mosley, Dr. Rangaraj Satyapal, Ms Elizabeth Stretton, Dr. Helen Venning, Dr. Kishore Warrier (Queens Medical Centre, Nottingham); Dr. Beverley Almeida, Ms Katie Arnold, Ms Laura Beard, Ms Virginia Brown, Dr. Raquel Campanilho‐Marques, Ms Elli Enayat, Ms Yvonne Glackin, Ms Elizabeth Halkon, Dr. Nathan Hasson, Ms Audrey Juggins, Ms Laura Kassoumeri, Ms Sian Lunt, Ms Sue Maillard, Dr. Kiran Nistala, Dr. Clarissa Pilkington, Ms Stephanie Simou, Dr. Sally Smith, Ms Hemlata Varsani, Dr. Lucy Wedderburn (Great Ormond Street Hospital, London); Dr. Kevin Murray (Princess Margaret Hospital, Perth, Western Australia, Australia); Dr. John Ioannou, Ms Linda Suffield (University College London Hospital, London); Dr. Muthana Al‐Obaidi, Ms Sam Leach, Ms Helen Lee, Ms Helen Smith (Sheffield's Children's Hospital, Sheffield); Ms Emma Inness, Ms Eunice Kendall, Mr. David Mayers, Dr. Nick Wilkinson (Oxford University Hospitals, Oxford); Dr. Jacqui Clinch, Ms Helen Pluess‐Hall (Bristol Royal Hospital for Children, Bristol).
